# Variation of peripheral pulse transit time with internal vascular pressure changes induced by arm movement

**DOI:** 10.3389/fnins.2023.1121902

**Published:** 2023-02-06

**Authors:** Zhiwei Sun, Xinge Jiang, Hua Wu, Feifei Liu

**Affiliations:** ^1^School of Information Science and Electrical Engineering, Shandong Jiaotong University, Jinan, China; ^2^School of Science, Shandong Jianzhu University, Jinan, China

**Keywords:** arterial blood pressure, pulse transit time, hydrostatic principle, arm position, arterial properties

## Abstract

Pulse transit time (PTT) and blood pressure (BP) are widely used to quantify arterial characteristics. Arm position influences arterial BP and peripheral PTT. This study aims to quantify the relationship between PTT changes with internal vascular pressure variations induced by the arm moving. With left arm at horizontal position as reference and the right arm moving from 90 to 45, 0, −45, and −90° respectively, PTT difference was calculated by the difference of the pulse foot between right arm and left arm within the same heartbeat. The change in the BP was calculated from the gravitational effect with the measured arm length. Our results showed that the change in PTT with arm elevating is more obvious than that with arm lowering, indicating the different relationship between PTT changes due to the internal BP changes. This can help in understanding the inherent physiological/pathological mechanism of cardiovascular system.

## 1. Introduction

A common method to reflect the performance of the cardiovascular system includes extraction of the information contained in radial artery pressure waveforms (Liu et al., 2013, 2014). We used arterial blood pressure (BP) as an important indicator to reflect the performance of arteries. Previous studies demonstrated that the value of BP measurement was influenced by the arm position. Netea et al. (2003), Fouladi et al. (2018), and Pan et al. (2019) showed that the BP values recorded with the left arm above and below the level of the right atrium decreased with the lifting of the arm but increased with the lowering of the arm, which was explained to be the effect of hydrostatic forces (Merendino, 1961; Webster et al., 1984). However, recent studies showed that the hydrostatic theory is not the only explanation for the change in BP along the arm. Gavish and Gavish (2011, 2013) found that the changes in both systolic blood pressure (SBP) and diastolic blood pressure (DBP) showed high linear correlations with the length of the arm. However, the systolic rate is lower than the diastolic rate with the right arm lifting.

The PTT is a widely-used measurement for quantifying arterial properties (Zheng and Murray, 2011; Mol et al., 2020). Recently, PTT is also used as a new technique for BP measurement (Atef et al., 2017; Cho and Park, 2021). The relationship between BP and PTT has been widely studied. Pereira (Schaanning and Skjaervold, 2020) reported that the PTT deduced from different locations can be used to measure BP by studying the relationship between BP and PTT in the static arm. Patzak et al. (2015) concluded that PTT showed a linear relationship in the low BP ranges while it showed an exponential relationship in the high BP ranges in the situation where the BP increased with the intravenous administration of dobutamine. Liu and Zhang (2006) studied the relationship between the PTT variations and the changes in the BP induced by the right arm lifting. The authors also reported that the PTT increased and the radial mean arterial pressure (MAP) gradually decreased with the arm lifting. Foo et al. (2005) studied the PTT changes induced by the different limb positions, in which the right or left arm was randomly selected for lifting and/or the right or left leg was randomly selected for lowering. Their studies showed that the mean PTT value increased with the arm lifting, while the mean PTT value decreased with the leg lowering. Previous studies showed that different arm position involves different muscles or muscles in different states. The relationship between PTT and BP may be different because of arm lifting or lowering (Siu et al., 2016). To make this clearer, we investigated the relationship between the changes in the PTT and the changes in the internal vascular pressure induced by arm movement.

## 2. Methods

### 2.1. Volunteers and setting

According to the Declaration of Helsinki (1989) by the World Medical Association, 22 healthy volunteers (13 men and 9 women) aged between 21 and 46 years were recruited from Shandong Jiaotong University, Jinan, China. The study received ethical permission from the Research Ethics Committee of the Affiliated Hospital of Shandong First Medical University, China (No. 2022001). All subjects provided their written informed consent.

The design of this study has been described in detail in our previous study (Jiang et al., 2017). Briefly, the design of the study is that the left arm was placed at the horizontal position as a reference and the right arm was moved at five different positions (90, 45, 0, −45, and −90°). To reduce the impact of intravascular physiological changes caused by the acute setting of arm position change, there were two identical repeat sessions in the whole measurement process for radial artery pulses. In one session, the measurement position sequence of the moving arm was 90, 45, 0, −45, and −90°, whereas, in the other session, the sequence was −90, −45, 0, 45, and 90°. At each session, the radial artery pulses were recorded when the signals were stable at each position (mostly, the moving arm was held at each position for 10–20 s before the measurement). Then, 10 successive radial artery pulse segments were obtained simultaneously from both arms. SBP and DBP were measured at the beginning and the end of the study and then MAP was calculated. Each subject's arm length (the distance between the clavicle and the radial artery) was measured and listed in Table 1.

**Table 1:** Arm length of the 22 subjects studied.

**Subject's no**.	**1^#^**	**2^#^**	**3^#^**	**4^#^**	**5^#^**	**6^#^**	**7^#^**	**8^#^**	**9^#^**	**10^#^**	**11^#^**
Arm length (cm)	64.6	66.5	69.0	66.5	65.0	68.0	65.7	71.0	66.9	65.4	65.4
Subject's no.	12^#^	13^#^	14^#^	15^#^	16^#^	17^#^	18^#^	19^#^	20^#^	21^#^	22^#^
Arm length (cm)	67.0	69.0	64.2	62.4	69.0	62.4	65.0	63.2	58.9	61.6	63.6

^#^The sequence number of the subject.

### 2.2. Calculation of MAP changes and PTT differences

In this study, the difference between the half value of the arm length (the distance between the clavicle and the radial artery) and 10 cm (the distance from the clavicle to the shoulder) was considered as each subject's midpoint of the arm. The actual changes in blood pressure induced by the arm movements were calculated individually for each subject by using the lowering/lifting vertical distance from the midpoint of the arm and the hydrostatic principle (Zheng and Murray, 2009). For example, if the arm length measured from the clavicle to the radial artery was 64 cm, the effective arm length was considered 54 cm and the midpoint of the arm was the point on the effective arm 27 cm. With the arm lowering and lifting at 90 and 45°, respectively, the lowering/lifting vertical distances from the midpoint of the arm were 27 and 19.1 cm with increasing/decreasing blood pressure at 19.4 and 13.7 mm Hg, respectively.

**Figure 1:**
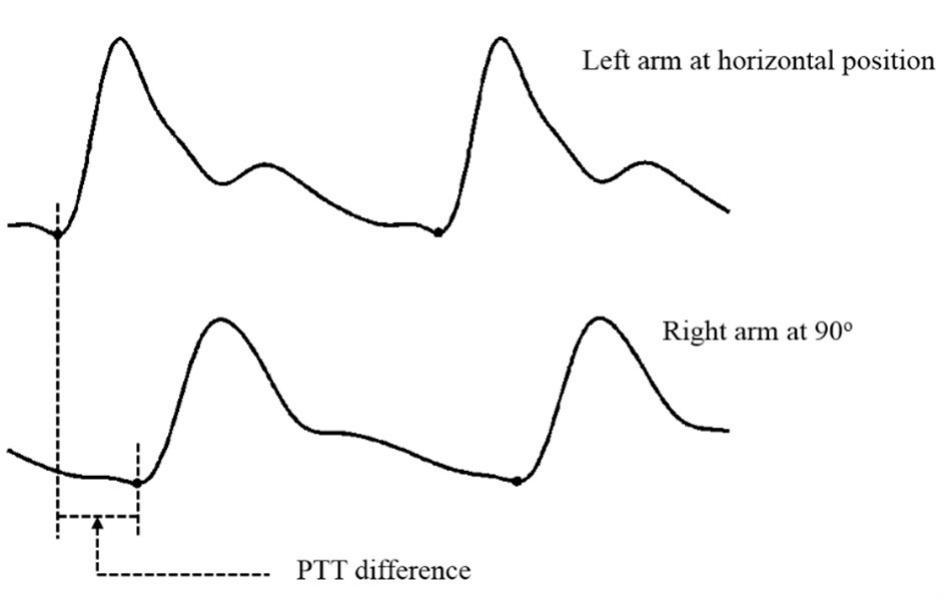
An example of calculating the PTT difference with the left arm at the horizontal position as a reference and the right arm at 90°.

PTT difference is equal to the difference in the pulse felt between the right arm and the left arm within the same heartbeat. As an example of calculating the PTT difference, Figure 1 shows the PTT difference with the left arm at the horizontal position as a reference and the right arm at 90°. Each subject's PTT differences at each position were calculated from 10 consecutive heartbeats.

The paired *t*-test was used to examine the effect of arm position on blood pressure and PTT, and a *p* < 0.05 was considered statistically significant.

## 3. Results

### 3.1. Mean blood pressure and PTT differences at different positions

**Figure 2:**
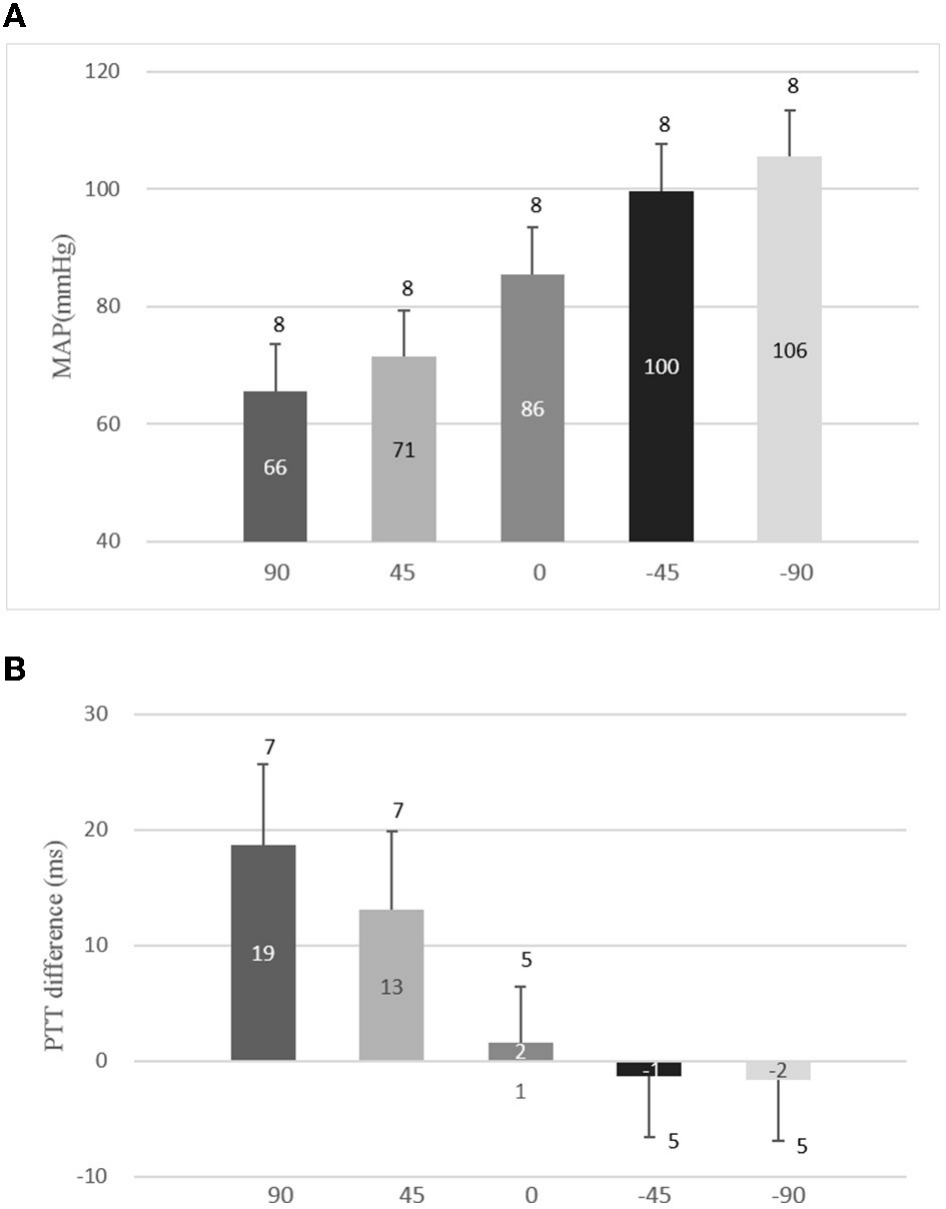
Means and standard deviations (SDs) of mean arterial pressure (MAP) and pulse transit time (PTT) differences with the right arm at different positions. **(A)** Means and SDs of MBP with the right arm at five positions to the horizontal level and **(B)** means and SDs of PTT differences with the right arm at five positions to the horizontal level.

Table 2 presents the overall means and standard deviations (SDs) of MAP and PTT differences with the right arm at five positions. The paired *t*-test was performed with a PTT difference of the non-horizontal level and a PTT difference of the horizontal level, respectively. Figure 2A shows the means and SDs of MBP with the right arm at five positions to the horizontal level. The changes in the arm position caused a significant effect on BP (all *p* < 0.01), and the variations of MAP were large with the arm below the horizontal level while small with the arm above the horizontal level (Figure 2A). Figure 2B shows the means and SDs of the PTT differences with the right arm at five positions to the horizontal level. The arm position caused a significant effect on PTT (*p* < 0.01 at 90, 45, −45, and −90°; *p* > 0.05 at 0°), which implies that the PTT differences were large with the right arm above the horizontal level while small with the right arm below the horizontal level.

**Table 2:** The overall means and SDs of MAP and PTT differences with the right arm at five positions.

**Position**	**90^o^**	**45^o^**	**0^o^**	**−45^o^**	**−90^o^**
MAP ( mm Hg)	66 ± 8^*^	71 ± 8^*^	86 ± 8	100 ± 8^*^	106 ± 8^*^
PTT differences (ms)	19 ± 7^*^	13 ± 7^*^	2 ± 5	−1 ± 5^*^	−2 ± 5^*^

^*^Significant statistical difference compared with 0°.

### 3.2. PTT changes with different MAP induced by various arm positions

**Figure 3:**
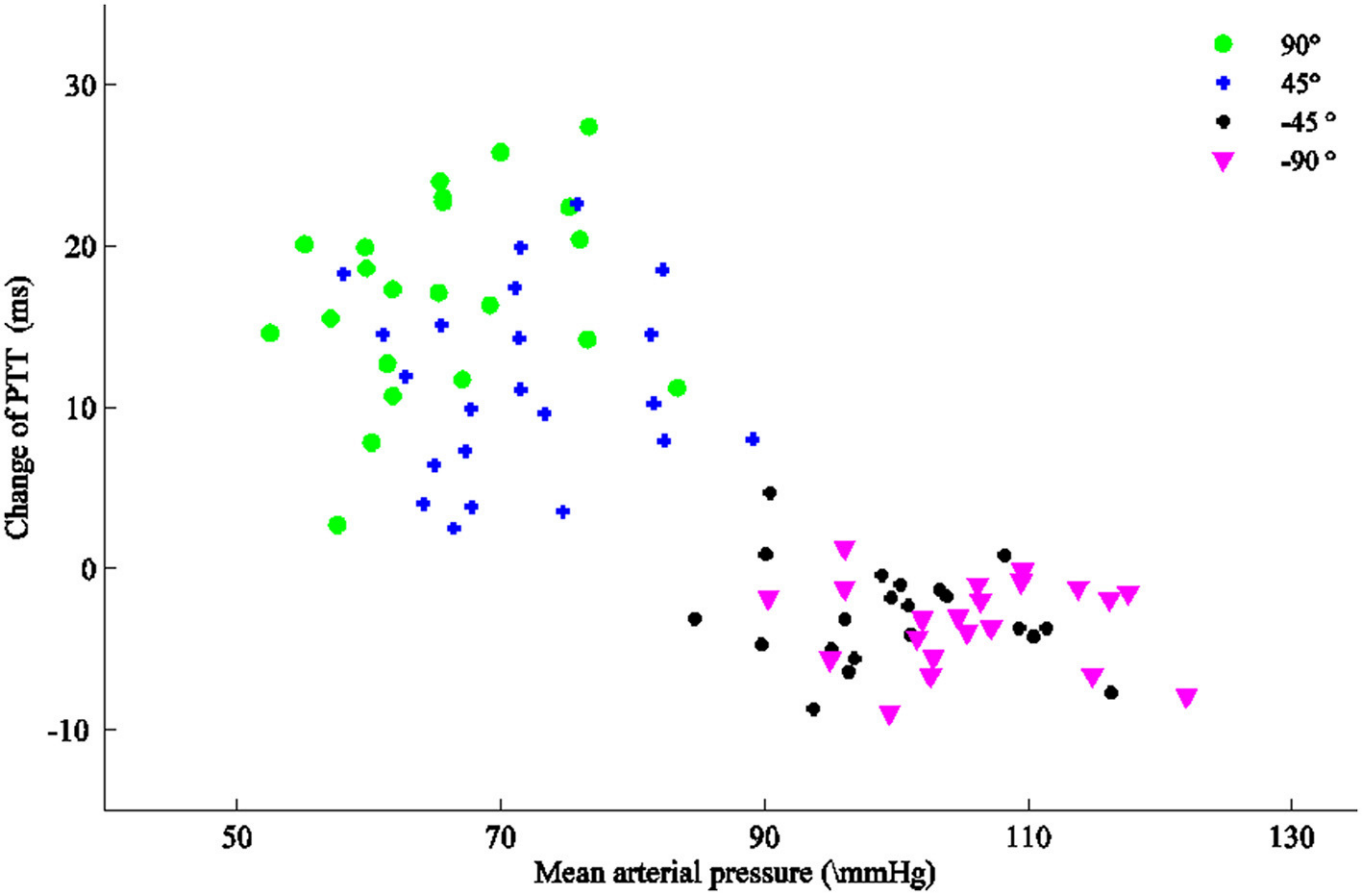
Relationship between each subject's changes in the pulse transit time (PTT) and corresponding mean arterial pressure (MAP) with the arm at 90, 45, −45, and −90^o^, respectively.

The changes in the PTT were calculated from the PTT differences of the right arm at positions 90, 45, −45, and −90° min those at 0°, respectively. The relationship between the changes in the PTT and a corresponding MAP with the arm positioned at 90, 45, −45, and −90° are depicted in Figure 3. With the arm moving at 90, 45, −45, and −90°, the changes in PTT were 17.1 ± 6.1, 11.4 ± 5.8, −3.0 ± 3.0, and −3.3 ± 2.6 ms and decreased, while the changes in MBP were 65.6 ± 8.0, 71.4 ± 8.0, 99.7 ± 8.0, and 105.5 ± 8.0 mm Hg and increased. Table 3 lists the rate of the means and SDs of the changes in the PTT and the linear regression equations with the right arm moving from 90 to −90° for each subject. As the rate was calculated with difference between the value with the right arm at a higher position and the value with the right arm at a lower position, the mean rates of the changes in the PTT shown in Table 3 were negative. However, if the rate was calculated with the difference between the value with the arm at a lower position and the value with the arm at a higher position, the rate shown in Table 3 would be positive. In this study, absolute values of the rate were used to identify the relationship between the changes in PTT and changes in the MAP induced by right arm movement. Table 3 points that the rates of changes in the PTT with the arm above the heart level (from 90 to 45° and from 45 to 0°) are larger (1.19 and 0.79) than those (0.21 and 0.06) with the arm below the heart level (from 0 to −45^o^ and from −45 to −90°).

**Table 3:** The means and SDs of the rate of changes in the PTT (ms) and linear regression equations with the right arm moving from 90 to −90° for each subject.

**Position**	**From 90°to 45°**	**From 45°to 0°**	**From 0°to −45°**	**From −45°to −90°**
Mean ± SD 90% CI	−1.19 ± 0.60	−0.79 ± 0.39	−0.21 ± 0.23	−0.06 ± 0.27
	(−2.17, −0.20)	(−1.43, −0.14)	(−0.59, 0.16)	(−0.51, 0.39)
Position	90°	45°	−45°	−90°
Linear regression equation	*y* = 0.21*x*+3.29 (*R*^2^ = 0.076)	*y* = 0.04*x*+8.78 (*R*^2^ = 0.003)	*y* = −0.07*x*+3.86 (*R*^2^ = 0.031)	*y* = −0.03*x*−0.27(*R*^2^ = 0.008)

## 4. Discussion

We investigated the relationship between the changes in the PTT and the changes in the MAP induced by the right arm lifting and lowering, and we found that (1) when the arm was positioned at 45 and 90°, the changes in the PTT increased while the BP decreased and (2) when the arm was positioned at −45 and −90°, the changes in the PTT increased and the BP increased. These results indicated that there were different relationships between PTT variations and MAP changes induced by different right arm positions. At 90° and 45°, the changes in the PTT were 17.1 and 11.4 with a rate of −1.19 ± 0.60 and −0.79 ± 0.39, respectively, while, at −45° and −90°, the changes in the PTT were −3.0 and −3.3 with the rate of −0.21 ± 0.23 and −0.06 ± 0.27, respectively.

Our results are consistent with the studies of Foo et al. (2005) and Liu and Zhang (2006), which showed that the changes in PTT increased and the BP decreased during the arm lifting. However, the research of Liu and Zhang only involves arm lifting and not arm lowering. Foo et al. also studied the relationship between PTT and BP induced by limb lifting and lowering. Lifting of one of the arms or lowering of one of the legs was used in the study of Foo et al., while we used the same arm to study the relationship between the changes in PTT and the changes in the BP induced by the right arm liftingor lowering. We found that there are different relationships between PTT changes and BP changes with the arm lifting or lowering at the same height relative to the level of the right atrium. With the arm lowering from the same height, the change in the PTT is smaller than that of the arm lifting. Zheng et al. (2007) and Zheng and Murray (2009) also indicated the relationship between internal vascular pressure and PTT with the arm at a different position. The data from Zheng and Murray's study are also in agreement with our data although they were not clearer.

This distinct result indicates that the effects of the physiological structure on PTT are different when lifting or lowering the arm. The reason may be partly due to different muscle states corresponding to different movements (Siu et al., 2016).

This study has several limitations. First, the sample involved only 22 subjects. The small sample size limits obtaining statistically significant results for a wide population. Second, when calculating the length of the midpoint of the arm, it was not accurate that 10 cm is considered to be the distance from the clavicle to the shoulder for all subjects, and the values of MBP with the arm positioned at 90, 45, −45, and −90° were estimated by the linear hydrostatic principle using the midpoint of the arm but not to the direct measuring. These values will cause some errors in calculating the changes in MBP. Finally, this study only involved healthy people and did not involve the diseased population.

## 5. Conclusion

This study demonstrated that there are different relationships between changes in PTT and changes in BP induced by right arm lifting or lowering at the same height relative to the level of the right atrium. Changes in PTT were larger during the arm lifting than the arm lowering with the approximate linear change in MBP. Future research should focus on the relationship between changes in PTT and changes in BP induced by arm movement for patients with a certain disease, which may thus be potentially useful for clinical applications.

## Data availability statement

The raw data supporting the conclusions of this article will be made available by the authors, without undue reservation.

## Ethics statement

The studies involving human participants were reviewed and approved by the Medical Ethics Committee of Jinshoulder, Lumbar, and Leg Pain Hospital Affiliated to Shandong First-Medical University. The patients/participants provided their written informed consent to participate in this study.

## Author contributions

ZS is responsible for data acquisition, programming, and thesis writing. XJ is responsible for experimental scheme design, data sorting, and paper modification. HW is responsible for the design of experimental equipment and the revision of papers. FL is responsible for guiding the experimental procedure. All authors contributed to the article and approved the submitted version.

## References

[B1] Atef M. Xiyan L. Wang G. Lian Y. (2016). “PTT based continuous-time non-invasive blood pressure system,” in IEEE International Midwest Symposium on Circuits and Systems (IEEE), 1–4. 10.1109/MWSCAS.2016.7870022

[B2] Cho H. S. Park Y. J. (2021). Measurement of pulse transit time using ultra-wideband radar. Technol. Health Care 29, 859–868. 10.3233/THC-202626 33427703 PMC8543252

[B3] Foo J. Y. Wilson S. J. Williams G. R. Harris M. A. Cooper D. M. (2005). Pulse transit time changes observed with different limb positions. Physiol. Meas. 26, 1093. 10.1088/0967-3334/26/6/018 16311456

[B4] Fouladi B. Joshi H. Edgell H. (2018). Cardiovascular and autonomic responses to passive arm or leg movement in men and women. Eur. J. Appl. Physiol. 119, 551–559. 10.1007/s00421-018-4030-9 30446863

[B5] Gavish B. Gavish L. (2011). Blood pressure variation in response to changing arm cuff height cannot be explained solely by the hydrostatic effect. J. Hypertens. 29, 2099–2104. 10.1097/HJH.0b013e32834ae315 21873886

[B6] Gavish B. Gavish L. (2013). Simple determination of the systolic-diastolic pressure relationship from blood pressure readings taken at different arm heights. Blood Press. Monit. 18, 144. 10.1097/MBP.0b013e328361c8fd 23635485

[B7] Jiang X. Wei S. Zheng D. Liu F. Zhang S. Zhang Z.. (2017). Change of bilateral difference in radial artery pulse morphology with one-side arm movement. Artery Res. 19, 1–8. 10.1016/j.artres.2017.04.008

[B8] Liu C. Tao Z. Zhao L. Chang F. Liu C. Wei S.. (2014). Modelling arterial pressure waveforms using Gaussian functions and two-stage particle swarm optimizer. Biomed Res. Int. 2014, 923260. 10.1155/2014/923260 24967415 PMC4054788

[B9] Liu C. Zheng D. Murray A. Liu C. (2013). Modeling carotid and radial artery pulse pressure waveforms by curve fitting with Gaussian functions. Biomed. Signal Process. Control. 8, 449–454. 10.1016/j.bspc.2013.01.00324211907

[B10] Liu Y. Zhang Y. (2006). Pulse Transit Time and Arterial Blood Pressure at Different Vertical Wrist Positions. Available online at: https://citeseerx.ist.psu.edu/document?repid=rep1&type=pdf&doi=848f9debbc19a7be9f3995c3e695863370c7db12

[B11] Merendino J. (1961). Importance of the position of the arm on the level of arterial blood pressure. JAMA. 175, 51. 10.1001/jama.1961.63040010010015c 13769586

[B12] Mol A. Meskers C. Niehof S. P. Maier A. B. van Wezel R. J. A. (2020). Pulse transit time as a proxy for vasoconstriction in younger and older adults. Exp. Gerontol. 135, 110938. 10.1016/j.exger.2020.110938 32247853

[B13] Netea R. T. Lenders J. P. Thien T. (2003). Both body and arm position significantly influence blood pressure measurement. J. Hum. Hypertens. 17, 459–462. 10.1038/sj.jhh.1001573 12821952

[B14] Pan F. He P. Chen F. Pu X. Zhao Q. Zheng D. (2019). Deep learning-based automatic blood pressure measurement: evaluation of the effect of deep breathing, talking, and arm movement. Ann. Med. 51, 397–403. 10.1080/07853890.2019.1694170 31724891 PMC7877882

[B15] Patzak A. Mendoza Y. Gesche H. Konermann M. (2015). Continuous blood pressure measurement using the pulse transit time: comparison to intra-arterial Measurement. Blood Press. 24, 217–221. 10.3109/08037051.2015.1030901 25857601

[B16] Schaanning S. G. Skjaervold N. K. (2020). Rapid declines in systolic blood pressure are associated with an increase in pulse transit time. PLoS ONE. 15, 1–16. 10.1371/journal.pone.0240126 33031455 PMC7544103

[B17] Siu A. Schinkel-Ivy A. Drake J. D. (2016). Arm position influences the activation patterns of trunk muscles during trunk range-of-motion movements. Hum. Mov. Sci. 49, 267–276. 10.1016/j.humov.2016.07.010 27522643

[B18] Webster J. Newnham D. Petrie J. C. Lovell H. G. (1984). Influence of arm position on measurement of blood pressure. Br. Med. J. 288, 1574–1575. 10.1136/bmj.288.6430.1574 6426648 PMC1441228

[B19] Zheng D. Allen J. Murray A. (2007). Non-invasive in vivo assessment of changes in peripheral arterial properties with estimation of arterial volume compliance. Physiol. Meas. 28, 1317. 10.1088/0967-3334/28/10/015 17906397

[B20] Zheng D. Murray A. (2009). Non-invasive quantification of peripheral arterial volume distensibility and its non-linear relationship with arterial pressure. J. Biomechanic. 42, 1032–1037. 10.1016/j.jbiomech.2009.02.011 19345360

[B21] Zheng D. Murray A. (2011). Peripheral arterial volume distensibility: significant differences with age and blood pressure measured using applied external pressure. Physiol. Meas. 32, 499. 10.1088/0967-3334/32/5/001 21422513

